# Radiation therapy in the era of immune treatment for hepatocellular carcinoma

**DOI:** 10.3389/fimmu.2023.1100079

**Published:** 2023-01-20

**Authors:** Lingjuan Chen, Ruiguang Zhang, Zhenyu Lin, Qiaoyun Tan, Zhiyong Huang, Binyong Liang

**Affiliations:** ^1^ Cancer Center, Union Hospital, Tongji Medical College, Huazhong University of Science and Technology, Wuhan, China; ^2^ Hepatic Surgery Center, Department of Surgery, Tongji Hospital, Tongji Medical College, Huazhong University of Science and Technology, Wuhan, China

**Keywords:** radiation therapy, immune checkpoint inhibitor (ICI), combination therapy, tumor microenevironment, hepatocellula carcinoma

## Abstract

Immune checkpoint inhibitors (ICIs) have revolutionized cancer treatment in recent years and provide new opportunities to treat hepatocellular carcinoma (HCC). To date, several ICIs have been approved by the FDA for advanced HCC in first-line or second-line therapy. Downstaging conversion therapy for potentially resectable HCC to provide opportunities for surgical intervention is challenging. ICIs have become a hot spot in this field due to their high response rate. However, HCC has various etiologies and can evade the immune system through multiple mechanisms, which limit the efficacy of ICI monotherapy and demand novel combination strategies. Radiation therapy (RT) is also a candidate for conversion therapy in HCC and is currently gaining increasing attention as a good combination partner with ICIs due to its ability to modulate the tumor microenvironment. In this review, we illustrate the current indications for ICIs and RT in HCC, the rationale for their synergistic combination, and the current clinical trials in combination therapy. We also speculate on predictive biomarkers and novel future strategies to further enhance the efficacy of this combination. This review aims to provide references for future research on radiation and immunotherapy to arrive at a promising new era of HCC treatment.

## 1 Introduction

Hepatocellular carcinoma (HCC) is one of the leading causes of cancer-related death globally (1). According to clinical practice guidelines and consensus (2–7), including the EASL and ESMO guidelines (2, 3), the standard treatments for early tumors include resection, liver transplantation, and local ablation, and those for intermediate-stage tumors include transarterial chemoembolization (TACE) and systemic drugs. Although several breakthroughs have occurred, the median overall survival is only 20~30 months for intermediate stages and 10~19 months for advanced-stage HCC (8). At the time of diagnosis, approximately 64% of HCC patients are identified with stage B or C disease [Barcelona Clinic Liver Cancer (BCLC)], losing the opportunity for radical hepatectomy (9, 10). Therefore, novel treatment approaches, especially conversion therapy for unresectable HCC patients, are desperately needed in the clinic.

Downstaging conversion therapy for potentially resectable HCC to provide opportunities for surgical intervention is challenging. HCC was proven to be an immunosuppressive microenvironment and related to inflammation (11). The expression of programmed death-ligand 1 (PD-L1) correlates with prognosis in resected HCC patients (12). The upregulation of PD-1 and PD-L1 is associated with more advanced stages and higher recurrence risks (13), which suggests the benefit of immunotherapy in HCC patients. Currently, several immune checkpoint inhibitors (ICIs), including anti-PD1 (nivolumab, pembrolizumab, and nivolumab plus ipilimumab) and anti-PD-L1 (atezolizumab, in combination with bevacizumab) therapies, have received FDA approval and provided new opportunities to treat advanced HCC (14).

Radiotherapy is a promising treatment for unresectable HCC, with a local control rate of 60%~100% at 2 years (15–17). Radiation can induce immunogenic cell death, promote the immune system (18), and reprogram the tumor microenvironment (19). The combination of radiation and immunotherapy offers better local tumor regression and systemic control than single treatments (20, 21), as reported in multiple tumor types (22, 23).

In this review, we will illustrate the current indications for immunotherapy and radiation, especially in the field of neoadjuvant or conversion therapy. Second, we will briefly summarize the current clinical trials in combination therapy and the combination scheme. Finally, we will speculate on predictive biomarkers and future research directions for immunotherapy.

## 2 Indications of immune checkpoint inhibitors in hepatocellular carcinoma

### 2.1 Immunotherapy for advanced HCC

#### 2.1.1 First-line therapy

The international multicenter phase III IMbrave150 study showed that atezolizumab plus bevacizumab significantly improved overall survival (19.2 vs. 13.4 months) and progression-free survival (6.9 months vs. 4.3 months) compared with sorafenib (24). Based on this research, atezolizumab plus bevacizumab was approved by the FDA as a first-line systematic treatment for unresectable HCC. The ORIENT-32 study was similar to research in China, in which sintilimab plus a bevacizumab biosimilar (IBI305) was approved as the first-line treatment for unresectable HCC by the National Medical Products Administration (NMPA) in 2021 (25). Several phase I/II clinical studies, such as the RESCUE study of camrelizumab plus apatinib (26) and KEYNOTE−524 study of pembrolizumab combined with lenvatinib, also demonstrated the efficacy and controllable safety of combined immunotherapy. The phase III study CheckMate 459 of single ICI nivolumab (NIVO) compared with sorafenib (SOR) did not achieve the primary endpoint OS but demonstrated improved safety, which also provided a choice for patients with contraindications for antiangiogenic targeted therapy.

#### 2.1.2 Second-line therapy

At present, a phase III clinical study of combined immunotherapy results for HCC second-line therapy is lacking. However, multiple phase II studies have shown that the combination of ICIs has some advantages over monotherapy. The main evidence included (1) single ICI treatment: nivolumab (CheckMate 040 study) (27), pembrolizumab (KEYNOTE 224 study) (27), camrelizumab (28), and terelizumab (RATIONALE 208) (2); ICIs combined with antiangiogenic targeted therapy: camrelizumab combined with apatinib (RESCUE study) (26); and (3) ICI combination therapy: nivolumab (NIVO) + ipilimumab (CheckMate 040 study) (29).

### 2.2 Conversion therapy

Conversion therapy for HCC includes the conversion of surgically unresectable lesions, such as insufficient volume of the future liver remnant (FLR), to resectable lesions, and it supports the conversion of R1 and R2 resection to R0 resection. Specifically, conversion therapy is characterized by active treatment regimens with a relatively high response rate. Previous studies have shown that radiotherapy, interventional therapy, and targeted therapy can convert 11%~27% of advanced HCC with portal vein tumor thrombus (PVTT) into surgical resection (30–32). ICIs combined with antiangiogenic therapy improved response rates by approximately 24% to 46% with good conversion therapy potential (24, 26, 33). In particular, ICI-based therapy combined with radiotherapy for PVTT has become an important conversion strategy for advanced HCC.

### 2.3 Adjuvant immunotherapy

The 5-year recurrence rate after surgery for HCC is as high as 50%~70%, but a standard adjuvant treatment is lacking. ICIs used as adjuvant therapy for patients with high-risk recurrence of HCC are currently a research hotspot, and some phase III clinical studies are underway, including nivolumab (NCT03383458), pembrolizumab (NCT03867084), toripalimab (NCT03859128), atezolizumab combined with bevacizumab (NCT04102098), durvalumab alone or in combination with bevacizumab (NCT03847428), and camrelizumab combined with apatinib (NCT04639180).

## 3 Indications for radiotherapy in HCC at different stages

The purpose of radiotherapy can be divided into radical, palliative, consolidation or translational, and adjuvant (preoperative or postoperative) radiotherapy. Stereotactic radiotherapy for small HCC is aimed at radical treatment, while palliative radiation therapy is recommended for symptomatic primary HCC and/or tumor thrombus. For unresectable HCC, consolidative radiation is mostly after systemic therapy. Adjuvant radiation is recommended for resected IHC with high-risk features (34).

### 3.1 Stereotactic body radiation therapy of small HCC

Intrahepatic stereotactic body radiation therapy (SBRT) for liver cancer mainly targets small hepatocellular carcinomas (microtumors). A study comparing SBRT with radiofrequency showed that the 3-year overall survival rates were similar (70.4% versus 69.1%), but the 3-year recurrence rates were 5.3% and 12.9%, showing the local control advantage of SBRT (35).

### 3.2 Radiation before liver transplantation

SBRT is a safe and effective bridging therapy in the waiting period prior to transplantation for HCC patients. An analysis comparing SBRT and transcatheter arterial chemoembolization (TACE) and radiofrequency ablation used as bridging therapy before liver transplantation showed similar safety and efficacy (36).

### 3.3 Radiation as consolidation and neoadjuvant or conversion therapy

For HCC lesions larger than 5cm, with the double blood supply of the hepatic artery and portal vein, the portal venous blood supply remains intact after single TACE therapy, resulting in a residual tumor. Radiotherapy after TACE can compensate for the deficiency of TACE. A meta-analysis showed that the 3-year survival rates ranged from 24% to 44% for TACE combined with external radiotherapy, an increase of 10%~28% compared to TACE alone (37).

For patients with intermediate or advanced HCC, surgery following neoadjuvant or conversion therapy improved treatment efficacy (32, 38, 39). A meta-analysis that enrolled a total of 2577 patients with unresectable HCC showed that patients who received interventional therapy combined with radiotherapy had improved long-term survival compared with interventional therapy alone. The survival pooled odds ratios at 2, 3, 4, and 5 years were 1.55, 1.91, 3.01, and 3.98, respectively, showing that radiotherapy can significantly improve overall survival, especially long-term survival (40).

### 3.4 Radiation in HCC with portal vein/inferior vena cava tumor thrombus

Radiotherapy is recommended as neoadjuvant treatment with surgical resection for stage IIIA HCC with PVTT, and it also improved the local tumor control rate and prolonged survival in combination with TACE. A multicenter randomized controlled study enrolled resectable HCC patients with PVTT who were randomly divided into a preoperative neoadjuvant radiotherapy group (82 cases) and a single surgery group (82 cases). The preoperative radiotherapy dose was 18 Gy/6 F for the tumor and PVTT, and surgery was performed 4 weeks after radiotherapy. The results showed that the preoperative radiotherapy group had 1- and 2-year survival rates (75.2% and 27.4%, respectively) that were significantly higher than those in the simple surgical resection group (43.1% and 9.4%, respectively) (41). Another study compared TACE plus radiotherapy with sorafenib in HCC patients with tumor thrombus, the median overall survival was longer in the TACE + radiotherapy group than in the sorafenib group (55 weeks vs. 43 weeks, P=0.04), and PFS (30 weeks vs. 11.3 weeks, P<0.01) (31).

### 3.5 Adjuvant placement after narrow margin resection of HCC

For the majority of central HCC and a small number of peripheral HCC cases, the requirement of a surgical safety margin >1 cm is difficult to meet due to tumor proximity or the involvement of the hilar vascular trunk, even after surgical resection of the tumor. Some patients even have a positive margin, which limits the curative effect of surgery. The Chinese Academy of Medical Sciences reported the results of adjuvant radiotherapy after narrow margin resection of HCC for the first time (42). A total of 181 patients were enrolled, including 33 patients in the narrow-margin surgery combined with postoperative radiotherapy group (Group A), 83 patients in the narrow-margin surgery group who did not receive radiotherapy (Group B), and 65 patients in the wide-margin surgery group (Group C). The 3-year survival rates were 89.1%, 67.7% and 86.0%, respectively, and the 3-year disease-free survival rates were 64.2%, 52.5% and 60.1%, respectively. Furthermore, patients in Groups A and C experienced significantly fewer early recurrences (*P* = 0.002) and substantially fewer intrahepatic margins (*P* = 0.048), diffuse recurrences (*P* = 0.018), and extrahepatic metastases (*P* = 0.038) than patients in Group B, with no patient developing radiation-induced liver disease. This study preliminarily suggests that postoperative adjuvant radiotherapy can compensate for the lack of narrow-margin surgery.

### 3.6 Radiotherapy for extrahepatic metastases

External radiotherapy for HCC liver lymph node metastasis is safe and effective (43, 44). For adrenal gland metastasis (45), bone or soft tissue metastasis (46), lung metastasis (47), and brain metastasis (48), radiotherapy can also reduce the size of metastases and relieve symptoms, which has clinical benefits.

## 4 Combination of RT and ICIs

### 4.1 The immune microenvironment of HCC

Most cases of HCC are due to chronic hepatitis infection, which is characterized by a chronic inflammatory state (49). Chronic HBV infection is the main risk factor for HCC in Southeast Asia and Africa, whereas chronic infection with hepatitis C is the main risk factor for HCC in Western countries and Japan (50–54). Among patients with HCC in the United States, approximately 50% to 60% are infected with HCV, 10% to 15% are infected with HBV, approximately 20% to 25% have alcoholic liver disease, and approximately 20% to 30% of patients with HCC have some features of metabolic syndrome (55). Chronic hepatitis infection perturbs the liver microenvironment and tips the scales in favor of carcinogenesis (49). The tumor microenvironment (TME) of HCC is a complex mixture of hepatic nonparenchymal resident cells, tumor cells, immune cells, and tumor-associated fibroblasts. Cytokines and chemokines secreted in the liver can promote angiogenesis, anti-apoptotic responses, and immune evasion, which facilitates an immunosuppressive microenvironment and promotes tumor growth (56, 57). Some studies suggested that the objective response rate (ORR) for PD-1/PD-L1 inhibitors did not significantly differ between virally infected and uninfected HCC patients, the tumor mutational burden was similar between the two groups of patients, and a difference was not observed in the ORR between HBV-HCC and HCV-HCC patients (58, 59).

### 4.2 Rationales for the combination of RT and ICIs

ICIs have recently revolutionized cancer treatment, while the overall response rates of single ICI therapy are only approximately 20%, which needs further improvement. Several rationales support RT as one of the most encouraging combination strategies. Specifically, RT can augment both antigenicity and adjuvanticity in addition to altering the local TME, which are critical for the immune response. First, radiation induces cell death, which results in cytosolic DNA accumulation, and enhances antigen release in the tumor to activate the production of type I interferon (IFN) genes *via* the cGAS/STING pathway (60–62). Second, RT promotes the release of tumor antigens and danger-associated molecular patterns (DAMPs), activates antigen-presenting cells (APCs), such as dendritic cells (DCs), and primes T cells within draining lymph nodes (63, 64). Third, radiation helps tumor-infiltrating lymphocytes (TILs) overcome the physical barriers of a tumor and facilitates the adaptive immune response (20, 65, 66). Fourth, reactive oxygen species (ROS) generated during radiation can modify macromolecules, such as proteins and DNA, which increases antigenicity. The presence of oxygen and generation of ROS are critical for direct DNA damage and radiation-induced tissue injury (67). At the same time, RT could change the immunogenicity of tumors from low to high through the increased expression of MHC class I and FAS on tumor cells (66, 68, 69). In addition to the cGAS-STING pathway, the release of DAMPs and cytokines can enhance adjuvanticity, elicit the migration of the pro-anticancer immune subpopulation, alter the TME and tilt the immune response to cancer cell killing. Overall, radiation converts cancer cells into an *in situ* vaccine to elicit anticancer immunity (70).

**Figure 1:**
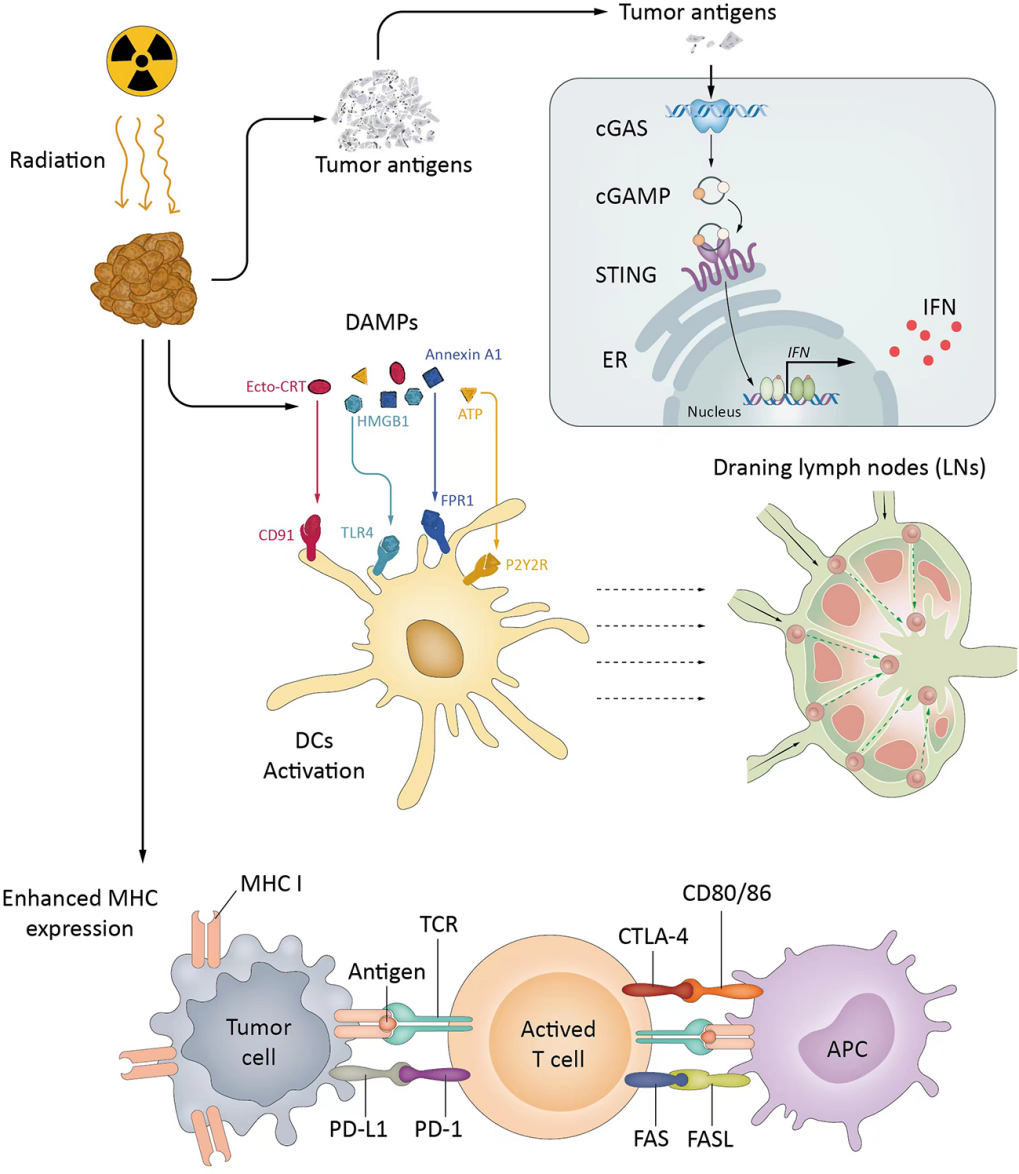
The synergistic effects of radiotherapy and ICIs. RT activates the cGAS/STING pathway, promotes APCs, and primes Tils into TME (cold to hot). RT increases MHC expression and changes the immunogenicity of tumors. ICIs reverse inhibitory signals and exhaustion pathways mediated by RT. cGAS, Cyclic guanosine monophosphate-adenosine monophosphate synthase; CTLA-4, Cytotoxic T lymphocyte-associated protein 4; IFN, Interferon; LN, Lymph node; MHC, Major histocompatibility complex; PD-1, Programmed death 1; PD-L1, Programmed death-ligand 1; STING, Stimulator of interferon genes; TAA, Tumor-associated antigen; TCR, T-cell receptor; Trex1, Three prime repair exonuclease 1.

Moreover, the addition of ICIs to radiation could help overcome radiation-induced immunosuppressive effects, and ICIs also enhance the RT-induced abscopal effect, referring to the regression of unirradiated tumors (71). Therefore, the synergistic effects of radiotherapy and ICI combinations are diversified (**Figure 1**).

### 4.3 Preclinical data and ongoing trials for HCC

Numerous preclinical studies and clinical settings have proven the synergistic effects of the combination of ICI and RT (iRT) in various cancer types (72–76). Several clinical studies have shown clinical benefits from iRT therapy. A meta-analysis including 20 clinical trials and 2,027 NSCLC patients showed that iRT therapy was associated with a significantly improved ORR and OS (77). Durvalumab has already been approved as maintenance therapy after chemoradiation therapy for stage III NSCLC patients based on the Phase III PACIFIC trial (78, 79). However, studies on the synergistic effect of HCC are still ongoing. Several preclinical studies have investigated the efficacy and mechanism of iRT in murine HCC models. Kim et al. (80) showed that the combination of anti-PD-L1 and 10 Gy RT significantly suppressed HCC tumor cell growth and improved the survival of tumor-bearing mice compared to those with anti-PD-L1 alone or RT alone, which occurred *via* IFN-γ/STAT3 signaling. Another preclinical study (81) supported the synergistic effect of the SBRT and anti-PD-1 combination in an orthotopic murine HCC model. The combination of SBRT (30 Gy in 3 fractions) and anti-PD-1 antibodies markedly suppressed tumor growth and improved survival with increased infiltration of CD8+ cytotoxic T cells within the tumor. Another study showed that the ionizing radiation (IR)-induced DNA damage repair (DDR) inhibitor AZD6738 in combination with RT and ICIs in HCC led to stronger immunologic memory, lasting antitumor immunity than radioimmunotherapy, and the prevention of tumor recurrence in mouse models, which relied on the activation of the cyclic GMP-AMP synthase/stimulator of interferon genes (cGAS/STING) signaling pathway (82). These data showed that the tumor response to radiation can be augmented by concurrent treatment with ICIs. These findings are promising and indicate the potential mechanisms by which combination therapy overcomes immune resistance to generate lasting immunity and protect against tumor recurrence.

In addition to encouraging data in preclinical studies, some published clinical data and case reports have shown promising results regarding the combination treatment of RT and ICIs in HCC. Chiang et al. reported a 100% ORR with SBRT followed by nivolumab for 5 patients with unresectable HCC (83). A phase I trial (NCT02239900) that evaluated liver/lung SBRT with ipilimumab reported that 23% of patients had clinical benefits (84). A retrospective cohort study showed that SBRT combined with a PD-1 inhibitor improves PFS and OS in TACE-refractory patients with intermediate-stage HCC (85). Wonmo Sung’s mechanistic mathematical model showed that for the ICI-RT treatment regimen, the irradiated tumor fraction is the most important parameter for the efficacy, and adding RT to ICI yields an increase in clinical benefit from 33% to 71% in nonirradiated tumor sites for 90% of the tumor cells being irradiated (86). One retrospective study showed that albumin-bilirubin (ALBI) scores and age were independent prognostic factors for PFS and OS in unresectable HCC treated with combined ICIs and RT (87). Radiation-induced liver disease (RILD) is the main adverse effect after liver radiation due to overexposure (88) and typically occurs 4–8 weeks after the termination of RT (89). The key point for preventing RILD is to maintain the liver within the tolerance range limit when designing the RT plan (89). The liver tolerance dose (average dose of the liver) is 23 Gy for Child−Pugh A patients and only 6 Gy for Child−Pugh B patients (90). The ASTRO Clinical Practice Guideline suggests that the selection of dose-fractionation regimen and technique should be based on disease extent, disease location, underlying liver function, and available technologies (34). These trials showed that the most common grade 3/4 treatment-related adverse reactions were elevated AST and ALT levels, colitis, or hand-foot skin reactions attributable to either radiation or immunotherapy, but no patient experienced grade 4 or 5 treatment-related toxicity. Most episodes of toxicity were transient and could be managed (83–85). These studies provide preliminary evidence to show that iRT is safe and efficacious, and they show that systemic immune activation was greater after liver irradiation. Several prospective clinical trials registered at www.ClinicalTrials.gov to evalute the combination of RT and ICIs in HCC are ongoing (**Table 1**).

**Table 1:** On-going clinical trials for a combination of immune checkpoint inhibitors and radiation.

NCT number	Institution	Phase	Disease	Intervention	Estimated enrollment	Primary endpoint
NCT03482102	United States (MGH)	II	Locally advanced/unresectable or metastatic HCC or biliary tract cancer	Tremelimumab + Durvalumab + EBRT	70	ORR
NCT03203304	United States (UCh)	I	Unresectable HCC	Nivolumab + SBRT vs nivolumab and ipilimumab + SBRT	50	Number of participants with adverse events
NCT03316872	Canada (UHN)	II	HCC progression after sorafenib	Pembrolizumab +SBRT	30	ORR
NCT04167293	China (Sun Yatsen University Cancer Center)	II/III	HCC with portal vein invasion	SBRT + sintilimab vs SBRT	116	6-mo PFS
NCT03817736	Hong Kong (Queen Mary Hospital)	II	HCC	TACE/SBRT + ICI	33	Number of patients eligible for curative surgical interventions
NCT04611165	South Korea (NCC)	II	HCC with major vascular invasion	Nivolumab + EBRT	50	PFS
NCT04547452	China (West China Hospital)	II	Metastatic HCC	SBRT + sintilimab vs sintilimab	84	PFS
NCT04193696	China (Guangxi Medical University)	II	Advanced HCC	RT+ anti-PD-1 agent	39	ORR

MGH, Massachusetts General Hospital; HCC, Hepatocellular carcinoma; EBRT, External beam radiotherapy; UCh, University of Chicago; SBRT, Stereotactic body radiotherapy; UHN, University Health Network; PFS, Progression-free survival; TACE, Transcatheter arterial chemoembolization; ICI, Immune checkpoint inhibitor; NCC, National Cancer Center; ORR, Overall response rate; RT, Radiotherapy; PD-L1, Programmed cell death ligand 1.

## 5 Challenges with the combination approach

Although previous studies have demonstrated the efficacy and tolerable toxicity of RT and ICI combination therapy in HCC patients, an optimal RT dose, fractionation scheme, and RT/ICI sequencing have not been specified, and these parameters may depend on the treatment purpose, choice of ICIs, mutational burden and patient performance status (91).

### 5.1 Treatment sequences

ICIs can be administered before, after or even concurrently with RT. Several studies have been performed to determine the optimal sequences of RT and ICIs. The PACIFIC trial showed that durvalumab started within 14 days improved PFS after completing RT (79). One report showed that administering ICIs 7 days after RT was less effective in enhancing OS than concurrent administration (92). Further studies are required to determine the optimal timing of RT to maximize benefits in HCC patients when combined with ICIs.

### 5.2 Radiation dose and fractionation

SBRT or hypofractionated RT is widely accepted to possibly be more immunogenic than conventional fractionated RT with 2 Gy per day. Dewan et al. reported that an 8 Gy × 3 regimen plus anti-CTLA-4 mAb showed better local tumor control and a systemic abscopal effect than two other regimens, 20 Gy × 1 and 6 Gy × 5 (20). A retrospective cohort observation even showed that proton beam radiotherapy (PBT) combined with anti-PD1/PD-L1 provides a sustained and high rate of local tumor control in the irradiation field and an excellent systemic therapeutic effect, resulting in overall tumor control and survival (93). Gaining an understanding of the effect of different fractionations on the immune response is important to improve the combination of RT and ICIs to increase their efficacy and administer personalized ICIs (63).

### 5.3 Biomarkers

Finally, appropriate candidates for iRT need to be selected to improve outcomes for HCC patients. PD-L1 expression, tumor mutation burden, and the infiltration of effector T lymphocytes are selectable predictive biomarkers for ICIs in some tumor types but have not been evaluated in HCC (94). The next-generation sequencing-based profiling of immune gene expression signatures, T-cell receptor repertoire, T-cell-inflamed gene expression, and the microbiome is on the way to help identify patients most likely to derive a therapeutic response with HCC (95), not only for predicting susceptibility to ICIs but also individual radiation sensitivity (96–98). Therefore, further efforts to identify appropriate biomarkers to better select patients with HCC who are suitable for iRT are needed.

## 6 Prospects

Although the introduction of ICIs in HCC has been notably behind that in other tumors, progress in HCC immunotherapy has still advanced. In the near future, ICI treatments might be found to increase the efficacy of locoregional and radical treatments for HCC. In particular, neoadjuvant or conversion therapy for resectable or nonresectable HCC patients will have the chance of paving the way for a significant drop in mortality rates in this fatal disease.

Ideally, to achieve this goal, efforts should be made to combine ICIs, including radiation, antiangiogenic agents, and tyrosine kinase inhibitors (TKIs). These efforts should also include the development of new immunotherapy agents, such as agonist immunostimulatory monoclonal antibodies, bispecific antibodies, and neoantigen vaccination. The identification of useful biomarkers for classifying sensitivity and resistance to individual agents or combinations is important for advanced personalized therapy.

## 7 Conclusions

Within the last decade, immunotherapy research has grown remarkably and changed the treatment paradigm for HCC. In addition to neoadjuvant or conversion therapy, ICIs have a well-established role in the advanced stage. Improvements in radiation have boosted the efficacy of RT in HCC. RT can remodel the “cold” TME to an immune-reactive “hot” TME, synergistically improving the effectiveness of ICIs. The development of synergistic combinations will probably be an important direction in the future. Along the way, the quest for accurate, user-friendly biomarkers will probably also be necessary for personalized immunotherapies.

## Author contributions

LC was involved in the writing and editing of the manuscript. RZ, ZL and QT participated in data and literature review. ZH and BL were responsible for the study conception and design. All authors contributed to the article and approved the submitted version.
